# Long-term impact of paediatric critical illness on the difference between epigenetic and chronological age in relation to physical growth

**DOI:** 10.1186/s13148-023-01424-w

**Published:** 2023-01-14

**Authors:** Ines Verlinden, Grégoire Coppens, Ilse Vanhorebeek, Fabian Güiza, Inge Derese, Pieter J. Wouters, Koen F. Joosten, Sascha C. Verbruggen, Greet Van den Berghe

**Affiliations:** 1 Clinical Division and Laboratory of Intensive Care Medicine, Department of Cellular and Molecular Medicine, KU Leuven, Herestraat 49, B-3000 Leuven, Belgium; 2 Intensive Care Unit, Department of Paediatrics and Paediatric Surgery, Erasmus Medical Centre, Sophia Children’s Hospital, Rotterdam, The Netherlands

**Keywords:** Critical illness, Children, PICU, DNA methylation, Epigenetic, Epigenetic age, Ageing, Growth, Development

## Abstract

**Background:**

Altered DNA-methylation affects biological ageing in adults and developmental processes in children. DNA-methylation is altered by environmental factors, trauma and illnesses. We hypothesised that paediatric critical illness, and the nutritional management in the paediatric intensive care unit (PICU), affects DNA-methylation changes that underly the developmental processes of childhood ageing.

**Results:**

We studied the impact of critical illness, and of the early use of parenteral nutrition (early-PN) versus late-PN, on “epigenetic age-deviation” in buccal mucosa of 818 former PICU-patients (406 early-PN, 412 late-PN) who participated in the 2-year follow-up of the multicentre PEPaNIC-RCT (ClinicalTrials.gov-NCT01536275), as compared with 392 matched healthy children, and assessed whether this relates to their impaired growth. The epigenetic age-deviation (difference between PedBE clock-estimated epigenetic age and chronological age) was calculated. Using bootstrapped multivariable linear regression models, we assessed the impact hereon of critical illness, and of early-PN versus late-PN. As compared with healthy children, epigenetic age of patients assessed 2 years after PICU-admission deviated negatively from chronological age (*p* < 0.05 in 51% of bootstrapped replicates), similarly in early-PN and late-PN groups. Next, we identified vulnerable subgroups for epigenetic age-deviation using interaction analysis. We revealed that DNA-methylation age-deceleration in former PICU-patients was dependent on age at time of illness (*p* < 0.05 for 83% of bootstrapped replicates), with vulnerability starting from 6 years onwards. Finally, we assessed whether vulnerability to epigenetic age-deviation could be related to impaired growth from PICU-admission to follow-up at 2 and 4 years. Multivariable repeated measures ANOVA showed that former PICU-patients, as compared with healthy children, grew less in height (*p* = 0.0002) and transiently gained weight (*p* = 0.0003) over the 4-year time course. Growth in height was more stunted in former PICU-patients aged ≥ 6-years at time of critical illness (*p* = 0.002) than in the younger patients.

**Conclusions:**

As compared with healthy children, former PICU-patients, in particular those aged ≥ 6-years at time of illness, revealed epigenetic age-deceleration, with a physical correlate revealing stunted growth in height. Whether this vulnerability around the age of 6 years for epigenetic age-deceleration and stunted growth years later relates to altered endocrine pathways activated at the time of adrenarche requires further investigation.

**Supplementary Information:**

The online version contains supplementary material available at 10.1186/s13148-023-01424-w.

## Background

Critical illness in children is defined as any life-threatening medical condition that requires support of failing vital organs to avoid imminent death and can be caused by a variety of insults such as major surgery, trauma, burn injury or severe medical illnesses. Although nowadays, the majority of critically ill children survive, they suffer from long-term consequences such as growth retardation, poor physical functioning and impaired neurocognitive and psychosocial development observed up to years after hospital discharge [1–3]. Part of this long-term legacy of paediatric critical illness appeared to be caused by the early administration of parenteral nutrition in the paediatric intensive care unit (PICU), with vulnerability determined by age [4–6]. In a recent epigenome-wide DNA-methylation (DNAm) study, we have shown that critical illness, and the early administration of parenteral nutrition, altered the methylation status of DNA extracted from leukocytes between PICU admission and discharge, which statistically explained (part of) the long-term developmental sequelae [7]. Subsequently, we also showed that the methylation status of DNA extracted from buccal mucosa swabs 2-years after PICU admission was altered and related to the long-term developmental legacy [8].

Altered DNAm has previously shown to affect biological ageing of adults and developmental processes in children [9, 10]. Environmental factors, such as physical activity and diet and exposure to drugs and toxins, have shown to alter the rate at which humans biologically age [9, 11–13]. Also major trauma, physical stress, chronic systemic inflammation and metabolic dysregulation affect the ageing process [9, 10, 14]. We therefore hypothesised that critical illness in children also affects the epigenetic changes underlying the ageing process, which during childhood predominantly reflects physical growth.

Biological age is predictable from DNAm profiles by machine learning models, such as the ones developed by Horvath and Hannum, models that were labeled “epigenetic clocks” [15, 16]. Recently, an epigenetic clock specifically for children has been developed, the PedBE clock [17]. The PedBE clock model, which is freely accessible as open source software [17, 18], is based on epigenome-wide DNAm profiling of DNA extracted from 1721 buccal mucosa swabs obtained from 11 different cohorts of typically developing children aged 0 to 20 year old. This clock selected 94 CpG sites, using ElasticNet penalised regression, as most predictive for chronological age. As such, this clock mapped healthy developmental patterns during childhood. Hence, the difference between the PedBE-estimated age and the chronical age of children can serve as a biomarker of altered developmental trajectories [17, 19]. With use of the PedBE algorithm, we here investigated whether paediatric critical illness and its nutritional management affect the epigenetic age of children in relation to their long-term impaired physical growth.

## Methods

### Study population, data acquisition and ethical approval

This study is a preplanned secondary analysis of patients who participated in the multicentre PEPaNIC randomised controlled trial that included 1440 critically ill children aged 0–17 years admitted to the paediatric intensive care units (PICUs) of Leuven (Belgium), Rotterdam (The Netherlands) or Edmonton (Canada) and its 2- and 4-year follow-up studies in comparison with matched healthy children [4, 5, 20]. The patients in this study had been randomly assigned to early initiation of parenteral nutrition within 24 h when enteral nutrition was insufficient (“early-PN”), or to postponing any supplemental PN to beyond the first week in PICU (“late-PN”). After one week, for both groups equally, PN could be administered if necessary [20]. The full study protocol has been published [21].

Weight and height, assessed in former PICU-patients 2 and 4 years after inclusion in the PEPaNIC RCT, was compared with equivalent longitudinal measurements in matched healthy children, who had not previously been admitted to a neonatal ICU or PICU, or to a hospital for 7 days or more with need of an intravenous line [4, 5]. Apart from unrelated healthy children, also siblings and relatives of the patients were included, to control as much as possible for genetic and socio-economic/environmental background. At the 2-year follow-up, after obtaining written informed consent from parents and/or the children, buccal mucosa swabs (Isohelix, Cell Projects, Harrietsham, Kent, England) were collected following the standard collection procedure. Swabs were stored in a DNA stabilising solution (DSK kit, Isohelix) at -80 °C until further processing.

All PEPaNIC patients and matched healthy controls from whom a buccal mucosa swab was available were eligible for the DNAm analyses and for the analysis of physical growth.

The institutional review boards at each participating site approved this follow-up study (ML8052; NL49708.078). The study was performed in accordance with the 1964 Declaration of Helsinki and its amendments.

### DNA extraction and DNAm data processing

DNA was extracted from all available buccal mucosa swabs from patients and healthy children (DDK DNA isolation kit, Isohelix). DNA concentrations were quantified with the Qubit^®^ 3.0 fluorometer (Thermo Fisher Scientific, Waltham, MA). 200 ng DNA was subsequently subjected to bisulfite-conversion with use of the EZ-96 DNA Methylation-Direct^®^ Kit (Zymo Research, Irvine, CA). Bisulfite-converted DNA was processed using the Infinium^®^ HumanMethylation EPIC BeadChip (Illumina Inc., San Diego, CA). Data were subsequently processed using R statistical software version 3.5.3 [22]. The data were subjected to several consecutive quality assessments at sample and probe level. First, samples not showing the typical bi-peak curve of the methylation value distribution in the low- and high-end range on the sample histograms (density plots) were excluded. Beta-values (ranging from 0 or no methylation to 1 or full methylation) and corresponding M-values (log2 ratios of the intensities of methylated probes versus unmethylated probes) were then obtained from the raw intensities after background subtraction, colour correction and functional normalisation [23]. Next, to ensure that signals were expressed above the background defined by negative control probes, probes with a detection *p*-value greater than 0.01 in 50% of the samples were removed (*N* = 82) [23–25]. Also probes spanning known single nucleotide polymorphisms or located on sex chromosomes (*N* = 49,733) were removed [23–25]. Finally, a principal component analysis (PCA) on the M-values with *p*-value heatmap assessed the possible presence of non-biological or technical variation in the data (“batch effect”, Additional file 1; Additional file 2) [24]. Such non-biological or technical variation due to experimental conditions was corrected for by including the first 30 principal components (PCs) of the positive control probes located on the Infinium^®^ HumanMethylation EPIC BeadChip as covariates in all multivariable linear regression models [26]. In addition, we aimed to estimate the fraction of epithelial cells to evaluate potential cell heterogeneity among the samples. To this end, we made use of the EpiDISH algorithm [27], with the limitation that this algorithm has been developed for the Illumina 450 k array and about 5% of the CpG-sites used in this algorithm were not interrogated on the EPIC BeadChip that we used.

### Impact of paediatric critical illness and its nutritional management on the deviation between epigenetic and chronological age at 2-year follow-up

We first calculated the PedBE clock estimated epigenetic age for all former PICU-patients (early-PN and late-PN patients) and for healthy control children [17]. This clock makes use of 94 CpG sites, which all passed quality control in our sample set. We next plotted the univariate regression lines (with 95% confidence intervals) between chronological age and estimated epigenetic age, to detect any possible difference between healthy children and former PICU-patients, and between former patients randomised to early-PN and late-PN, across the age range of the population.

Next, the univariate findings were further analysed adjusting for risk factors. To this end, we calculated the difference between the PedBE clock-estimated epigenetic age and the chronological age (DNAm age minus chronological age), further referred to as “DNAm age-deviation”, for all former PICU-patients and healthy children. A positive DNAm age-deviation or “DNAm age-acceleration” reflects an “older” epigenome than the chronological age, whereas a negative DNAm age-deviation or “DNAm age-deceleration” reflects a “younger” epigenome than the chronological age. Differences in DNAm age-deviation between former PICU-patients and healthy children, and between early-PN and late-PN patients, were analysed with use of multivariable linear regression models, adjusting for the known baseline risk factors [age upon PICU admission, centre, gender, race, geographical origin, history of malignancy and a predefined syndrome (Additional file 3) and further for risk of malnutrition, severity of illness upon PICU admission and diagnosis group for the comparison between early-PN and late-PN group] and for technical variation in the DNA methylation data processing as explained above, with assessing interaction *p*-values for each baseline risk factor separately. For robustness, all analyses were repeated in 100 bootstrapped replicates, with results declared “robust” when significant associations were present in at least 50 replicates [28, 29].

### Assessment of physical growth from PICU admission, to follow-up at 2 and 4 years, in comparison with healthy children, as a possible correlate of the epigenetic findings

To assess whether any finding at the epigenetic level had a growth correlate, longitudinal growth in height and weight from PICU admission, to 2-years and 4-years follow-up was documented for former PICU-patients and over a similar time course for matched healthy children. Comparisons were done with multivariable repeated measures ANOVA assessing the interaction between groups and time and further with any relevant baseline risk group identified via the epigenetic analyses above. These subgroup analyses were only performed when a statistically significant interaction was identified.

### Data presentation and statistical significance and software

Data are presented as medians and IQRs, means and SDs, β estimates and 95% CI or numbers and proportions, as appropriate. The reported *p*-values are for the individual covariate resulting from a linear multivariable regression analysis in which 2-sided significance tests are used. Two-sided *p*-values less than 0.05 were considered statistically significant. Statistical analyses were done in JMP© version 15.0.0 (SAS Institute, Inc, Cary, NC) and R version 3.5.3 [22]. The LICMEpigenetics package version 0.1.0 was used to preprocess the epigenetic data [23]. This package contains R functions to exclude low-quality samples and probes, normalise the methylation data, adjust for batch effect, and find differentially methylated positions and regions, based on functions within the Minfi pipeline [8, 23, 30, 31].

## Results

**Fig. 1:**
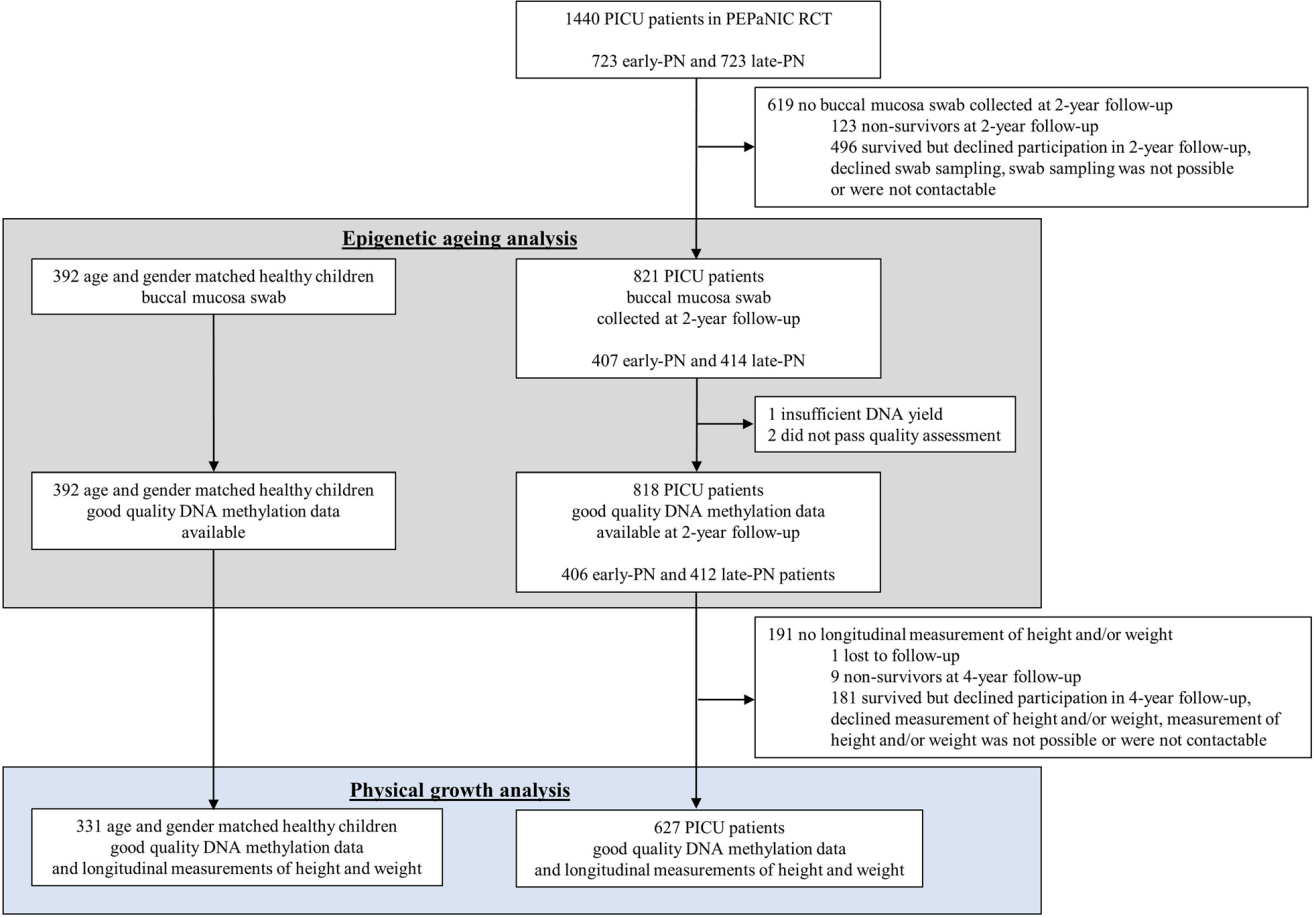
CONSORT diagram of study participants. Participants included in the epigenetic ageing analysis are indicated in grey. Participants included in the additional analysis of physical growth are indicated in blue. PICU: paediatric intensive care unit, PEPaNIC: Paediatric Early versus Late Parenteral Nutrition in Intensive Care Unit, RCT: randomised controlled trial

Good quality DNA from buccal mucosa was available for 818 patients, 406 randomised to early-PN and 412 to late-PN, and for 392 matched healthy children for assessment of the difference between epigenetic and chronological age at 2-year follow-up (Fig. 1; Table 1). The median estimated fraction of epithelial cells was comparable between patients (92%) and matched healthy children (93%). For 627 of these patients and 331 of these matched healthy control children, also longitudinal measurements of height and weight (upon PICU admission, at 2-years follow-up and at 4-years follow-up) were available (Fig. 1; Table 2).

**Table 1:** Demographics and medical characteristics of participants included in the epigenetic ageing analysis

Demographics and medical characteristics of participants	Healthy control children *N* = 392	PICU-patients *N* = 818	*p*-value	PICU-patients Early-PN *N* = 406	PICU-patients Late-PN *N* = 412	*p*-value
*Demographics*						
Age at 2-year follow-up (age at swab collection)						
Median (IQR)—years	3.8 (2.6–8.2)	3.4 (2.6–7.9)	0.96	3.5 (2.6–8.1)	3.2 (2.6–7.7)	0.60
Mean (SD)—years	6.0 (4.7)	6.0 (4.8)		6.0 (4.6)	6.0 (4.9)	
Gender						
Male—no (%)	212 (54.1)	475 (58.1)	0.19	238 (58.6)	237 (57.5)	0.75
Female—no (%)	180 (45.9)	343 (41.9)		168 (41.4)	175 (42.5)	
Known non-Caucasian race^*a*^—no (%)	32 (8.2)	66 (8.1)	0.95	39 (9.6)	27 (6.6)	0.10
Known non-European origin^*a*^—no (%)	51 (13.0)	144 (17.6)	**0.03**	82 (20.2)	62 (15.0)	**0.05**
*Medical characteristics*						
STRONGkids risk level^*b*^			/			0.69
Medium—no (%)	NA	736 (90.0)		367 (90.4)	369 (89.6)	
High—no (%)	NA	82 (10.0)		39 (9.6)	43 (10.4)	
PeLOD score, first 24 h in PICU^*c*^—mean (SD)	NA	21.0 (11.6)	/	21.0 (11.8)	21.1 (11.4)	0.69
PIM3 score^*d*^—mean (SD)	NA	−3.4 (1.4)	/	−3.4 (1.4)	−3.5 (1.3)	0.48
PIM3 probability of death^*e*^ (%)—mean (SD)	NA	6.7 (11.8)	/	6.9 (11.9)	6.4 (11.6)	0.63
Diagnostic category			/			0.71
Surg﻿ical						
Abdominal—no (%)	NA	63 (7.7)		28 (9.4)	29 (9.2)	
Burns—no (%)	NA	2 (0.2)		1 (0.3)	1 (0.3)	
Cardiac—no (%)	NA	364 (44.5)		126 (42.4)	138 (43.5)	
Neurosurgery-Traumatic brain injury—no (%)	NA	72 (8.8)		28 (9.4)	23 (7.3)	
Thoracic—no (%)	NA	41 (5.0)		20 (6.7)	15 (4.7)	
Transplantation—no (%)	NA	14 (1.7)		3 (1.0)	7 (2.2)	
Orthopaedic surgery-Trauma—no (%)	NA	40 (4.9)		12 (4.0)	7 (2.2)	
Other—no (%)	NA	26 (3.2)		8 (2.7)	13 (4.1)	
Medical						
Cardiac—no (%)	NA	24 (2.9)		8 (2.7)	14 (4.4)	
Gastrointestinal-Hepatic—no (%)	NA	5 (0.6)		1 (0.3)	1 (0.3)	
Oncologic-Haematologic—no (%)	NA	5 (0.6)		2 (0.7)	3 (1.0)	
Neurologic—no (%)	NA	49 (6.0)		16 (5.4)	19 (6.0)	
Renal—no (%)	NA	0 (0.0)		0 (0.0)	0 (0.0)	
Respiratory—no (%)	NA	78 (9.5)		30 (10.1)	34 (10.7)	
Other—no (%)	NA	35 (4.3)		14 (4.7)	13 (4.1)	
Malignancy—no (%)	0 (0.0)	39 (4.8)	** < 0.0001**	24 (5.9)	15 (3.6)	0.12
Syndrome^*f*^—no (%)	4 (1.0)	168 (20.5)	** < 0.0001**	81 (20.0)	87 (21.1)	0.67

Bold indicates a statistically significant difference between groups

^a^Participants were classified according to race and geographical origin by the investigators

^b^Scores on the Screening Tool for Risk on Nutritional Status and Growth (STRONGkids) range from 0 to 5, with a score of 0 indicating a low risk of malnutrition, a score of 1 to 3 indicating medium risk, and a score of 4 to 5 indicating high risk

^c^Paediatric Logistic Organ Dysfunction (PeLOD) scores range from 0 to 71, with higher scores indicating more severe illness

^d^Paediatric Index of Mortality 3 (PIM3) scores, with higher scores indicating a higher risk of mortality

^e^Paediatric Index of Mortality 3 (PIM3) probability of death

^f^A pre-randomisation syndrome or illness a priori defined as affecting or possibly affecting development (Additional file 3)

*IQR* interquartile range; *NA* not applicable; *no* number; *PeLOD* paediatric logistic organ dysfunction score; *PICU* paediatric intensive care unit; *PIM3* paediatric index of mortality 3 score; *PN* parenteral nutrition; *SD* standard deviation

**Table 2:** Demographics and medical characteristics of participants included in the physical growth analysis

Demographics and medical characteristics of participants	Healthy control children *N* = 331	PICU-patients *N* = 627	*p*-value
*Demographics*			
Age upon PICU admission			
Median (IQR)—years	1.7 (0.2–5.4)	1.5 (0.2–5.5)	0.97
Mean (SD)—years	3.5 (4.4)	3.7 (4.7)	
Age at 2-year follow-up			
Median (IQR)—years	3.5 (2.6–7.3)	3.3 (2.6–7.5)	0.66
Mean (SD)—years	5.6 (4.2)	5.8 (4.6)	
Age at 4-year follow-up			
Median (IQR)—years	5.4 (4.4–9.2)	5.2 (4.4–9.3)	0.83
Mean (SD)—years	7.5 (4.3)	7.7 (4.6)	
Gender			
Male—no (%)	178 (53.8)	370 (59.0)	0.15
Female—no (%)	153 (46.2)	257 (41.0)	
Known non-Caucasian race^*a*^—no (%)	23 (7.0)	48 (7.7)	0.68
Known non-European origin^*a*^—no (%)	39 (11.8)	105 (16.8)	**0.03**
*Medical characteristics*			
Height upon PICU admission—mean (SD)—cm	90.4 (35.0)	89.0 (36.5)	0.56
Weight upon PICU admission—mean (SD)—kg	16.1 (14.6)	15.4 (15.1)	0.47
STRONGkids risk level^*b*^			–
Medium—no (%)	NA	560 (89.3)	–
High—no (%)	NA	67 (10.7)	–
PeLOD score, first 24 h in PICU^*c*^—mean (SD)	NA	21.4 (11.4)	–
PIM3 score^*d*^ – mean (SD)	NA	-3.5 (1.3)	–
PIM3 probability of death^*e*^ (%)—mean (SD)	NA	6.3 (11.4)	–
Diagnostic category			–
Surgical			
Abdominal—no (%)	NA	49 (7.8)	
Burns—no (%)	NA	2 (0.3)	
Cardiac—no (%)	NA	287 (45.8)	
Neurosurgery-Traumatic brain injury—no (%)	NA	53 (8.5)	
Thoracic—no (%)	NA	33 (5.3)	
Transplantation—no (%)	NA	11 (1.8)	
Orthopaedic surgery-Trauma—no (%)	NA	30 (4.8)	
Other—no (%)	NA	22 (3.5)	
Medical			
Cardiac—no (%)	NA	21 (3.6)	
Gastrointestinal-Hepatic—no (%)	NA	3 (0.5)	
Oncologic-Haematologic—no (%)	NA	3 (0.5)	
Neurologic—no (%)	NA	38 (6.1)	
Renal—no (%)	NA	0 (0.0)	
Respiratory—no (%)	NA	48 (7.7)	
Other—no (%)	NA	27 (4.3)	
Malignancy—no (%)	0 (0.0)	34 (5.4)	** < 0.0001**
Syndrome^*f*^—no (%)	1 (0.3)	113 (18.0)	** < 0.0001**

Bold indicates a statistically significant difference between groups

^a^Participants were classified according to race and geographical origin by the investigators

^b^Scores on the Screening Tool for Risk on Nutritional Status and Growth (STRONGkids) range from 0 to 5, with a score of 0 indicating a low risk of malnutrition, a score of 1 to 3 indicating medium risk, and a score of 4 to 5 indicating high risk

^c^Paediatric Logistic Organ Dysfunction (PeLOD) scores range from 0 to 71, with higher scores indicating more severe illness

^d^Paediatric Index of Mortality 3 (PIM3) scores, with higher scores indicating a higher risk of mortality

^e^Paediatric Index of Mortality 3 (PIM3) probability of death

^f^A pre-randomisation syndrome or illness a priori defined as affecting or possibly affecting development (Additional file 3)

*IQR* interquartile range; *NA* not applicable; *no* number; *PeLOD* paediatric logistic organ dysfunction score; *PICU* paediatric intensive care unit; *PIM3* paediatric index of mortality 3 score; *SD* standard deviation

### Impact of paediatric critical illness and its nutritional management on the deviation between epigenetic and chronological age at 2-year follow-up

**Fig. 2:**
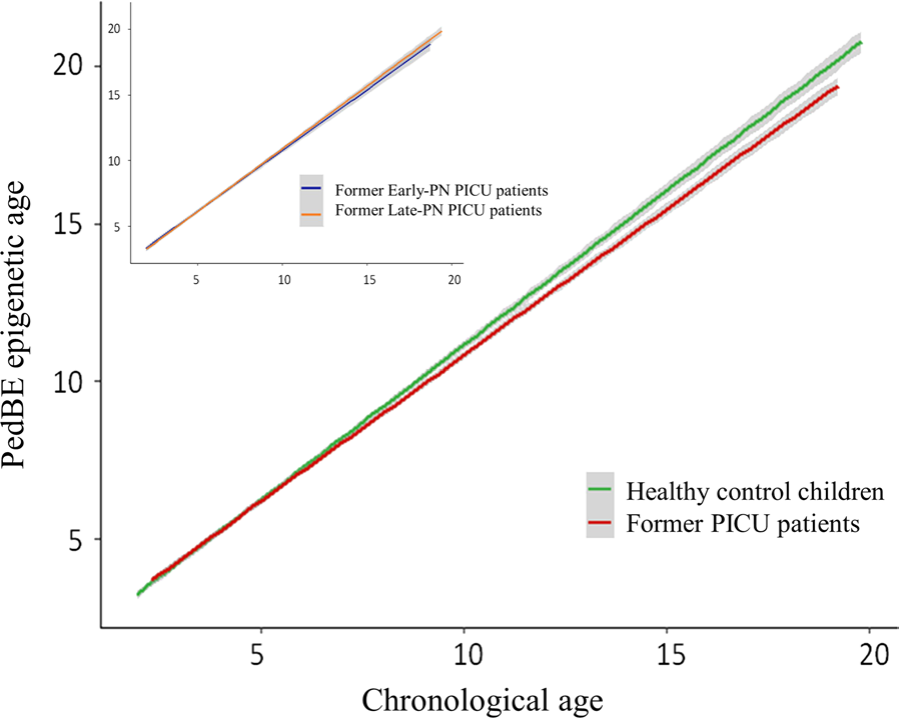
PedBE calculated DNAm age versus chronological age at 2-year follow-up—univariate plot. The chronological age at 2-year follow-up (x-axis) is plotted versus the PedBE estimated DNAm age (y-axis) for former PICU-patients and healthy control children. The red line indicates the linear regression for the former PICU-patients, the green line for the healthy control children. The grey zones indicate the 95% confidence interval. An increasing difference between former PICU-patients and healthy controls with age in the DNAm age-deviation was present. Separation of the curves started around the age of ± 8 years at 2-year follow-up (equivalent to ± 6 years at PICU admission). The inserted panel indicates the chronological age at 2-year follow-up (x-axis) versus the PedBE-estimated DNAm age (y-axis) for former early-PN versus late-PN PICU-patients. The blue line indicates the linear regression for the former early-PN PICU-patients, the orange line for former late-PN patients. The grey zones indicate the 95% confidence interval. No difference in DNAm age-deviation was present between former early-PN and late-PN PICU-patients. PICU: paediatric intensive care unit, PN: parenteral nutrition

The univariate regression analysis revealed that, as compared with healthy children, the estimated epigenetic age of former PICU-patients deviated negatively from the chronological age, starting from ± 8 years at 2-year follow-up onward (equivalent to ± 6 years at PICU admission), with former early-PN and late-PN patients behaving similarly (Fig. 2).

The multivariable linear regression analyses, adjusting for baseline risk factors, showed a significant DNAm age-deviation in former PICU-patients as compared with healthy children across the age range of the population in 51% of the bootstrapped replicates (Table 3). The bootstrapped multivariable analyses of interaction with baseline risk factors revealed that this DNAm age-deviation in former PICU-patients was depending solely on the age at time of critical illness (interaction *p* < 0.05 for 83% of the bootstrapped replicates). Together with the univariable regression lines, this multivariable adjusted analysis pointed to an age-dependent vulnerability for DNAm age-deviation starting from a chronological age of ± 6 years at time of critical illness onward (Table 3). Also in this multivariable analysis, there were no differences between early-PN and late-PN patients (data not shown).

**Table 3:** DNAm age-deviation in former PICU-patients as compared with healthy children

Analyses	Percentage of significant bootstrap replicates	Median *p*-value
Without interaction	51	0.04936
Interaction with age at PICU admission	83	0.00107
Interaction with race	28	0.16330
Interaction with origin	13	0.40436
Interaction with gender	11	0.41110
Interaction with centre	6	0.45385
Interaction with syndrome	2	0.63393

Summary of the bootstrapped multivariable linear regression models adjusted for baseline risk factors. The first model assessed whether a significant DNAm age-deviation was present in former PICU-patients as compared with healthy children, adjusted for baseline risk factors (first row). Next, for each baseline risk factor separately, an interaction term with patient-control status was additionally brought into the model (other rows). For each model, the percentage of significant bootstrap replicates is shown, as well as the median p-value of all bootstrap replicates

### Physical growth from PICU admission, to follow-up at 2 and 4 years, in comparison with healthy children, as a possible correlate of the epigenetic findings

**Fig. 3:**
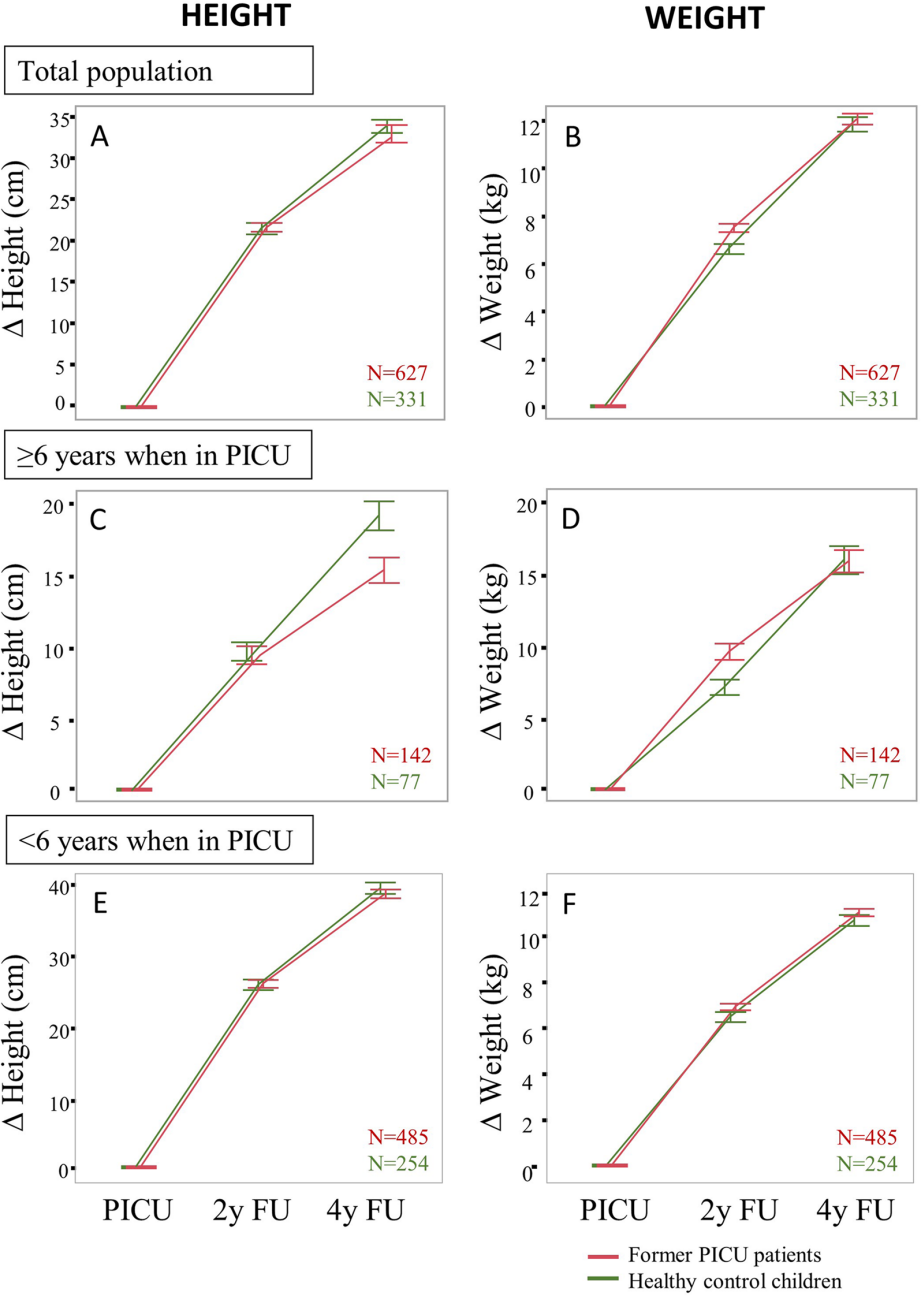
The 4-year time course of changes in height and weight for healthy children versus PICU-patients. Changes from baseline are presented as means and standard errors. Former PICU-patients are indicated in red and healthy control children in green. PICU-patients grew less in height, but transiently gained weight, as compared with matched healthy children over the 4-year time course (panels **A** and **B**). Growth in height was more stunted in PICU-patients ≥ 6 years of age at time of exposure to critical illness as compared with those aged < 6 years (panels **C** and **E**). Also, the transient weight gain in former PICU-patients was more present in those aged ≥ 6 years at time of exposure as compared with those aged < 6 years (panels **D** and **F**). ∆: delta, 2y FU: 2-year follow-up, 4y FU: 4-year follow-up, *N*: number, PICU: paediatric intensive care unit

The finding that former PICU-patients deviated epigenetically from their chronological age as compared with healthy children, starting from an age of ± 6 years at time of exposure to critical illness onwards, had a physical growth correlate (Fig. 3).

Multivariable ANOVA showed that former PICU-patients grew less in height (interaction *p* = 0.0002), but transiently gained weight (interaction *p* = 0.0003) (Fig. 3, panels A and B), as compared with matched healthy children over the 4-year time course. Given that the epigenetic data revealed vulnerability of former PICU-patients aged ≥ 6 years at time of exposure to critical illness, the impact on growth was also analysed, with age at time of critical illness dichotomised as ≥ 6 years versus < 6 years. This analysis confirmed that growth in height was more stunted in PICU-patients ≥ 6 years of age at time of exposure to critical illness as compared with those aged < 6 years (interaction *p* = 0.002) (Fig. 3, panels C and E). Especially from 2-year to 4-year follow-up, growth in height declined in the ≥ 6 year-old PICU-patients, deviating away from the healthy control children. Also, the transient weight gain in former PICU-patients was more present in those aged ≥ 6 years at time of exposure as compared with those aged < 6 years (interaction *p* < 0.0001) (Fig. 3, panels D and F).

## Discussion

We showed that former PICU-patients, and in particular those aged 6 years or older at time of exposure to critical illness, deviated epigenetically from their chronological age, revealing DNAm age-deceleration or a “younger” epigenome than that of matched healthy children. These findings had a physical correlate as the former PICU-patients showed stunted growth in height but not in weight, particularly in those aged 6 years or older at exposure. The use of early-PN versus late-PN during critical illness did not affect epigenetic ageing.

Whereas “ageing” in adults refers to the decline of biological processes culminating in death, “ageing” during childhood should be seen as growth and development. Early epigenetic clocks were trained exclusively or predominantly on samples obtained from adults and were thus designed to map this process of decline [17, 19, 32]. Hence, in adults, a DNAm age-acceleration has been associated with risk of age-related diseases and a DNAm age-deceleration with certain health benefits [13, 33–35]. These epigenetic clocks developed in adults are not well suited to map the processes of growth and development in childhood [32]. The recently developed PedBE clock circumvented this problem as it was trained on paediatric samples only [17]. With this paediatric clock, accelerated DNAm age-deviation reflects accelerated growth and development and a decelerated DNAm age-deviation reflects decelerated growth and development. We here observed DNAm age-deceleration in former PICU-patients as compared with matched healthy children, in particular in patients aged 6 years and older at time of the critical illness. We can only speculate on the reason why younger PICU-patients did not show this DNAm age-deceleration 2 years after critical illness. The epigenome of young children is known to have a high degree of plasticity, with most epigenetic remodeling taking place during the first 5 years after birth [36, 37]. Hence, it is possible that methylation changes at the 94 CpG sites used for the PedBE clock did occur during critical illness in younger children but were subsequently erased and thus no longer detectable 2 years later.

We also observed that the epigenetic age-deceleration observed in former PICU-patients coincided with stunted growth over the studied 4-year time interval. This is not unexpected given that the physical process most correlating with chronological ageing during childhood is growth in height. Our observation that also for the reduced growth in height, the former PICU-patients aged 6 years or older at time of critical illness were most vulnerable further supported the biological relevance of the epigenetic findings. The age of 6 years is also the age of onset of the adrenarche [38, 39]. The release of adrenal androgens at adrenarche, followed by a steep activation of the growth hormone axis and depending on a normal thyroid function, drives the mid-childhood growth spurt, which precedes the pubertal growth spurt [38]. Interestingly the genes related to the CpG sites included in the PedBE clock model are involved in these endocrine pathways [17]. In addition, these endocrine axes are profoundly disturbed by critical illness [40–42]. The finding that former PICU-patients only showed impaired growth in height but not weight, with even a transiently accelerated weight gain, is compatible with alterations in these hormonal axes.

The major strength of this study is the epigenetic and parallel longitudinal growth follow-up of large groups of former PICU-patients and demographically matched developing healthy children. The study also has a limitation; it remains unclear to what extent the epigenetic age-deceleration and the stunted growth observed in former PICU-patients were due to the acute critical illness and/or the intensive medical care, and to what extent factors and co-morbidities already present prior to PICU admission played a role. Although we did adjust all analyses for the known baseline risk factors, other important confounders may have remained unknown. Confirmation of the observed associations in other cohorts of critically ill children is warranted to strengthen these findings.

## Conclusion

We showed that, as compared with healthy children, former PICU-patients, in particular those aged 6 years or older at time of illness, revealed an epigenetic age-deceleration. These findings had a physical correlate revealing stunted growth in height. Whether this vulnerability around the age of 6 years for epigenetic age-deceleration and for stunted growth years later relates to altered endocrine pathways normally activated at the time of adrenarche requires further investigation.

## Supplementary Information

**Additional file 1. Title of data:** Quality assessment of the DNA methylation data: density plots. Description of data: Density plots indicate the frequency of beta-values over their full range (0 for no methylation to 1 for full methylation) for each sample.

**Additional file 2. Title of data:** Quality assessment of the DNA methylation data: principal component analysis. Description of data: Principal Component Analysis (PCA) is a technique used to bring out strong patterns in a dataset by reducing the dimensionality via creating new uncorrelated variables (principal components or PCs) that successively maximize variance. This p-value heatmap is a visualisation of the PCA, showing the association between the first eight PCs with biological (syndrome, patient vs control, origin, race, malignancy, gender, early-PN vs late-PN, centre, and chronological age) and non-biological factors (row on chip, plate, and chip).

**Additional file 3. Title of data:** Definition of ‘syndrome’. Description of data: This document describes the definition used to annotate ‘syndrome’ to a child.

## Data Availability

Data sharing is offered under the format of collaborative projects. Proposals can be directed to the corresponding author.
