# Supplementary material for: Long-term impact of paediatric critical illness on the difference between epigenetic and chronological age in relation to physical growth
Source: Clin Epigenetics. 2023 Jan 14;15:8. doi: 10.1186/s13148-023-01424-w (PMC9840263; doi:10.1186/s13148-023-01424-w)
Supplement: Supplementary file 1 — Additional file 1. Title of data: Quality assessment of the DNA methylation data: density plots. Description of data: Density plots indicate the frequency of beta-values over their full range (0 for no methylation to 1 for full methylation) for each sample. [file 13148_2023_1424_MOESM1_ESM.pdf]

Additional file 1. Quality assessment of the DNA methylation data: density plots

Panel A.

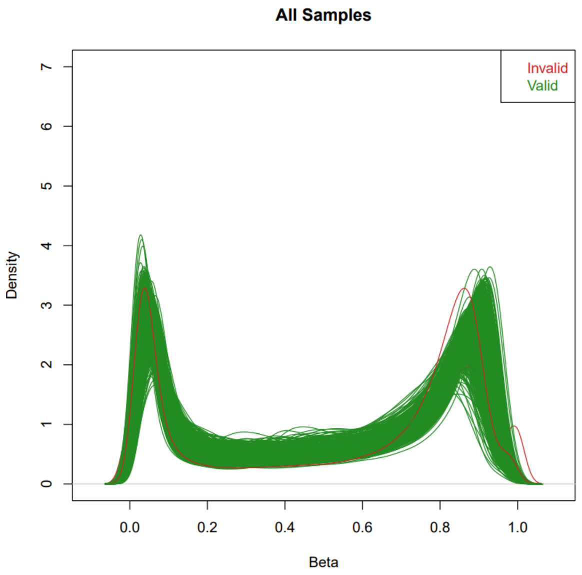

Panel B.

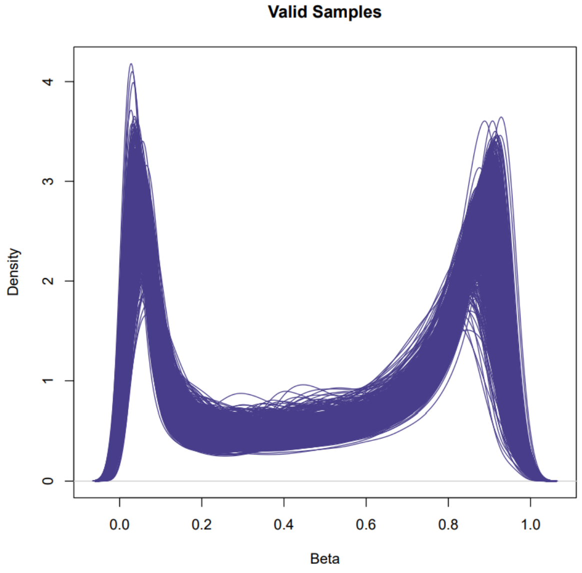

Density plots indicate the frequency of beta-values over their full range (0 for no methylation to 1 for full methylation) for each sample. In panel A, the frequency of the β-values of each sample is shown for all 1212 samples for which DNA yield was sufficient for DNA methylation analysis. Good quality samples are indicated in green and bad quality samples in red. The 1210 good quality samples are shown separately in Panel B. β-values, ranging from 0 (no methylation) to 1 (full methylation) are usually compressed in the low (between 0 and 0.2) and high (between 0.8 and 1) ranges. Thus, when β-value distribution among the samples is plotted, a bi-peak curve in the low- and high-end range is formed. If this is not the case or if the measured β-values follow a different trajectory as compared with the other samples, the measured β-values are not biologically plausible and probably due to bad sample quality (red lines Panel A).
