# Supplementary material for: Long-term impact of paediatric critical illness on the difference between epigenetic and chronological age in relation to physical growth
Source: Clin Epigenetics. 2023 Jan 14;15:8. doi: 10.1186/s13148-023-01424-w (PMC9840263; doi:10.1186/s13148-023-01424-w)
Supplement: Supplementary file 2 — Additional file 2. Title of data: Quality assessment of the DNA methylation data: principal component analysis. Description of data: Principal Component Analysis (PCA) is a technique used to bring out strong patterns in a dataset by reducing the dimensionality via creating new uncorrelated variables (principal components or PCs) that successively maximize variance. This p-value heatmap is a visualisation of the PCA, showing the association between the first eight PCs with biological (syndrome, patient vs control, origin, race, malignancy, gender, early-PN vs late-PN, centre, and chronological age) and non-biological factors (row on chip, plate, and chip). [file 13148_2023_1424_MOESM2_ESM.pdf]

Additional file 2: Quality assessment of the DNA methylation data: principal component analysis

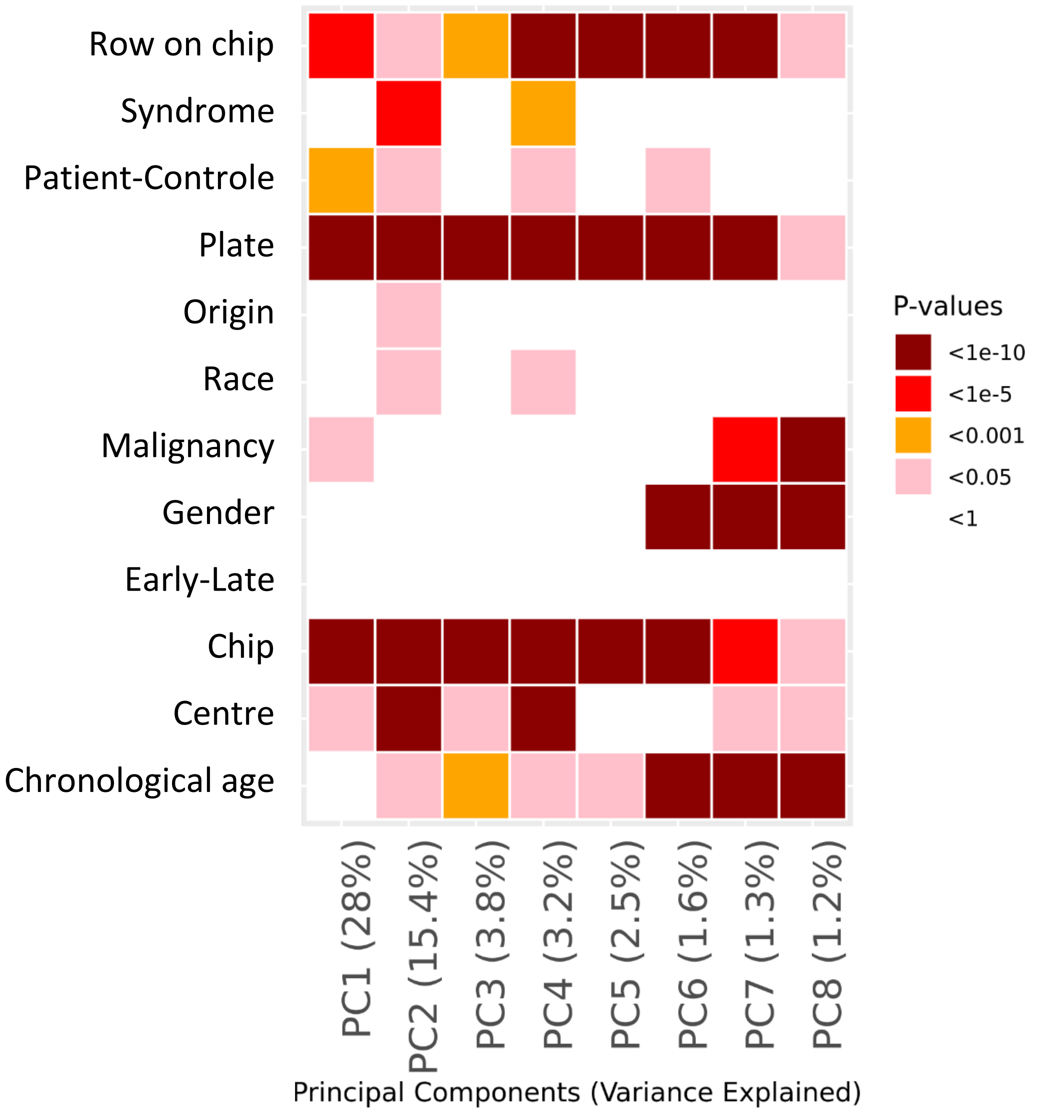

Principal Component Analysis (PCA) is a technique used to bring out strong patterns in a dataset by reducing the dimensionality via creating new uncorrelated variables (principal components or PCs) that successively maximize variance. This p-value heatmap is a visualisation of the PCA, showing the association between the first eight PCs with biological (syndrome, patient vs control, origin, race, malignancy, gender, early-PN vs late-PN, centre, and chronological age) and non-biological factors (row on chip, plate, and chip). An increasing red intensity indicates a stronger association between the two variables, based on the magnitude of the p-value of their association as assessed by ANOVA. The first three PCs explain 47.2% of the variance in the data. The heat map shows strong correlations between various principal components and the microarray chip, the well and plate on which a sample was run, which indicates that technical variation is present, warranting further correction.
