# Supplementary material for: Long-term impact of paediatric critical illness on the difference between epigenetic and chronological age in relation to physical growth
Source: Clin Epigenetics. 2023 Jan 14;15:8. doi: 10.1186/s13148-023-01424-w (PMC9840263; doi:10.1186/s13148-023-01424-w)
Supplement: Supplementary file 3 — Additional file 3. Title of data: Definition of ‘syndrome’. Description of data: This document describes the definition used to annotate ‘syndrome’ to a child. [file 13148_2023_1424_MOESM3_ESM.pdf]

Additional file 3. Definition of 'syndrome'

A prerandomisation syndrome or illness *a priori* defined as affecting or possibly affecting neurocognitive development, and which is subdivided in the following categories:

- Genetically confirmed syndrome or pathogenic chromosomal abnormality
- Clearly defined syndrome, association or malformation without (identified) genetic aberration
- Polymalformative syndrome of unknown aetiology
- Clear auditory or visual impairment without specified syndrome
- Congenital hypothyroidism due to thyroid agenesis
- Brain tumour or tumour with intracranial metastatic disease
- Paedopsychiatric disorder (e.g. autism spectrum disorder, (treatment for) attention deficit hyperactivity disorder)

- Severe medical disorder, not primarily neurologic, but suspected to alter psychomotor and/or mental performance
- Severe neonatal problem (e.g. severe asphyxia)
- Severe craniocerebral trauma or near-drowning
- Severe infectious encephalitis or drug-induced encephalopathy
- Infectious meningitis, encephalitis or Guillain-Barré
- Resuscitation and/or need for extracorporeal membrane oxygenation prior to randomisation
- Severe convulsions or stroke prior to randomisation.
